# Remarks on the Profession of Medicine in Sicily; an Exposition of the Principal Evils to Which It Is Subject in Great Britain; and Observations on Medical Reform

**Published:** 1815-09

**Authors:** John C. Yeatman

**Affiliations:** of Frome.


*For the London Medical and Physical Journal.*

### *Remarks on the Profession of Medicine in Sicily; an Exposition of the Principal Evils to which it is subject in Great Britain; and Observations on Medical Reform.* By Mr. JOHN C. YEATMAN, of Frome.

THE relations between this country and Sicily having afforded a ready communication with that celebrated island, we have been furnished with much information on its real state. As, however, the medical profession in Sicily has escaped the attention of modern writers, the following short view of that subject may not be altogether uninteresting.

The two universities at Catania and Palermo grant degrees in physic and surgery. The pupil is required to study three years before a degree is conferred on him, and he is not permitted to practise without it. The professors do not receive fees from their students, but a pittance of £30 sterling a-year from the funds of the university; the funds being made up of the fees for diplomas, which last amount to about £14 sterling each.

With the exception of a few physicians and surgeons educated at Pavia, Montpelier, and other places on the continent, the Sicilian faculty are, perhaps, a century behind the English in medical knowledge; they are unacquainted with modern authors, and their practice is chiefly confined to bleeding and certain sovereign remedies. They are miserably paid for professional attendance: a physician who receives two taris (tenpence currency) from one patient in five, thinks himself fortunate.

Midwifery is practised by women, the heat of the climate rendering parturition comparatively easy.

Whilst at Catania, on my tour to Ætna in the summer of 1810, I beheld for the first time the anatomical preparations of that university; they are all of wax, ingeniously and beautifully moulded, but, alas! how inaccurate. A gigantic figure of an adult, standing in a glass case, which bursts upon the view the moment the door of the Museum is opened, is only calculated to astound those who are unacquainted with anatomy. It is intended to delineate the vessels, nerves, muscles, viscera, &c. If a few minute's survey of this preparation enabled me to note the following inaccuracies and omissions, how numerous must they have proved upon a close examination.

The left common iliac vein passes directly under the artery throughout its whole course.

The femoral arteries are out of their usual direction.

 The

The superficial palmar arch lies close to the first phalanx of the fingers, and the radial and ulnar arteries contribute equally to its formation.

The anterior tibial artery branches off from the popliteal, and winds round the outer part of the leg under the common integuments before it finds its bed between the tibialis articus muscle and the bone.

The ulnar nerve gives two branches to the ring-finger.

The bladder is perched above the brim of the pelvis.

The sartorius and tensor vaginæ femoris are not to be seen in either thigh!

In a preparation of the brain, the external form is preserved, but the thalami, corpora striata, hippocampi, &c. are not to be discovered in the lateral ventricles.

I must not omit to mention the beautifully-wrought wax figure of a female in the advanced stage of utero-gestation, it shews the relative situation of parts with tolerable accuracy, by lifting up the abdominal muscles and the anterior part of the womb, which are so contrived as to let in and take off.

Whilst I was remarking that both ventricles of the model of a heart were of the same thickness, my fellow travellers hurried me away to the celebrated museum of the Prince of Biscari, or I had given a detailed account of these famous specimens of anatomical skill.

As the Sicilians are in a great measure ignorant of anatomy, so they are ill-acquainted with the nature and cure of disease, and the few who perform some of the secondary operations of surgery effect them in a very rude and mangling manner. If the foundation intended to build upon is slight or radically defective, the superstructure will be of no value. If the fountain is shallow, its numerous branches will receive but a scanty supply.

In a case of incised wound, wherein the brachial artery was partially divided, the vessel was seared with a red-hot iron, and the wound dressed with hot balsams. In a case of gunshot wound of the fore-arm, the stimulating balsams were also applied. This is their common practice. It is needless to mention the fatal termination of those cases.

A Benedictine monk received a blow by falling down the stairs of his convent, which deprived the deltoid muscle of its tone; and, although the limb could be moved about in any direction without pain, Sig. ———, the Professor of Surgery, pronounced it a dislocation of the head of the bone.

I recommended the removal of a small encysted tumour from the lower edge of the right pectoral muscle of a young child of good constitution; Sig. ——— had not done so,

 because because he believed the operation would be fatal to the patient.

If, however, the Sicilians have cause to lament the low state of medicine amongst them,—the Sicilians who labour under the blighting effects of an execrable government, which allows no liberty of the press, gives no encouragement to education, and impoverishes the people; how much more reason have we to regret, that in Great Britain, where the profession of medicine ranks so high,—a country which gave birth to a Harvey, a Sydenham, a Cullen, a Hunter, a Baillie, a Cooper, and an Abernethy,—hundreds of pretenders to physic and surgery should still be suffered to act in a profession than which none is more important and abstruse. "The mass of the medical faculty of the British empire is indeed an heterogeneous compound. Physicians, doctors of the English universities, fellows of the Royal College, versed in all human science, learned, honourable, and approximating to the "Corinthian capital of society," are pushed from their seats by doctors of no universities, unlearned men, and even women without science and without honour. The Abernethys, Coopers, Brookes's, have opposed to them self-created surgeons, raspers of shin-bones advertising gonorrhœa cures, and men whose chirurgical knowledge has been acquired by carrying a box after the dresser of an hospital. The legitimate apothecary is circumvented by the druggist who was yesterday a grocer, by the chemist who hardly knows a crucible from a cauliflower. To the educated physician, to the regular surgeon, to the instructed apothecary, this is, perhaps, personally unimportant; but what is it to the public?—pain, mutilation, death."* The empire "teems with this surreptitious multitude." "The druggists of a midland county, at this moment, are querulously complaining that a horrible attempt is making to take from them the privilege of prescribing for the sick."† But this is not all; a very large proportion of these practitioners, who are acting with the regulars of the medical body, are lamentably deficient in professional skill. A country apothecary who had the temerity to officiate in a case of strangulated hernia, begged to know of the surgeon (who judged it expedient to operate) what day he would fix for the operation! I lately saw, in a surgeon's surgery, the unimpregnated but thickened

* Note to the half-yearly Report of the Progress of Medicine for 1813, Medical and Physical Journal, vol. 30.

† Ibid.

womb of a young woman, which had been torn from the body by her medical attendant under the impression of its having been a fœtus! Another of these clever men sent a note to a surgeon with whom I was intimate, requesting "the lone of a pear of phorcepts" in a difficult case of labour! A few years ago, a practitioner brought together the torn muscles of a badly-lacerated wound of the fore-arm of a young man with two sutures; and, having placed a ligature upon a branch of the recurrens ulnaris (which, by-the-bye, he called the interosseal), put three sutures on the integuments; in twelve hours gangrene and death released the poor fellow from the agonizing pain attendant on the inflammation excited! A man, with a diploma from Aberdeen, administers Cayenne pepper, brandy, wine, bark, &c. in inflammatory fever! An old practitioner informed me, that the best way of increasing the flow of blood in arteriotomy, was to completely divide the vessel! Many men salivate for gonorrhœa and gleets; many use powerful injections during the inflammatory stage of that local disease; and others, conceiving all cases of spasm of the urethra cases of permanent stricture, have recourse to caustic bougies! A man in the lower part of this county, unrivalled for swearing and effrontery, professed to know every disease by examining the urine of the afflicted! —this fellow made a fortune with the water of his credulous patients; and, dying, the words "eminent apothecary" were inscribed on his monument. Near Kingswood, Gloucestershire, I was not a little amused with a sign embellished with a pestle and mortar, on which were the following words:—"I Popjay Surgeon Apotecary and Midwife &c. draws teeth and bleeds on the lowest terms. Confectionary Tobacco Snuff Tea Coffee Sugar and all sorts of Perfumery sold here. NB New laid Eggs every morning by Mrs. Popjay."

And at Blandford, in Dorsetshire:—

"I Biddiwood expert at deeds
I Periwigs makes
Draws teeth and bleeds."

A medical man once hit upon an ingenious method of concealing his professional ignorance from his medical neighbours; whenever they attempted to enter into a medical conversation with him, he invariably stifled it in its birth by saying, (if in the morning) "I never talk professionally before dinner;" and, (if in the afternoon,) "I never talk of the shop after dinner."

A charlatan honoured many places in this county with his presence last winter. With a long beard, a conjuror's dress, and a companion who was occasionally in livery; the sultan himself never appeared with more consequence in his divan than than did this impostor amidst his patients. He was to cure every complaint by the touch. Dr. Touch'em at last overshot the mark. He gave it out to the public that he would walk on the water from Rownham-Ferry Hotwells to Bristol Bridge; but, not performing his promise, was obliged to make a precipitate retreat amidst the hisses of coblers, tinkers, and chimney-sweepers.

Such are hundreds of persons who are permitted to tamper with and destroy the lives of the people. The legislature should in all ways exert its power for the preservation of public health; the profession of medicine should not be left open to ignorant persons: as life often depends upon the skill of the medical attendant, so it must be of the first importance that he should prove himself professionally competent to act in the above capacity, by undergoing an adequate examination before he is entrusted with the health and lives of the people. How are the lower orders of society, nay, the upper and most polished classes of persons, to detect the medical ignorance of the bombastic pretender to physic, of the grave self-important knave, or of the ever-smiling, smooth-speaking, hand-shaking, and party-giving practitioner? How disgraceful is it to the profession to find so many of its members afford, by their ignorance, an opportunity to Sir William Garrow, Mr. Jekyl, and others, to bring down the contempt of the court upon them in cases of medical jurisprudence, &c.

The facility with which ignorant persons creep by bye-paths into the profession of medicine appears to be the source of the numerous evils to which it is subject. Hence the pitiful jealousies towards each other, and incalculable mischief to society; hence the art and low-cunning resorted to to counterbalance the disadvantages arising from the want of education, talent, and interest; and, hence the "sarcasm of the wit," and the contempt of the scholar, so often directed against the whole medical body, though only felt by its unworthy members.

Every one insists on the necessity of medical reform, though many differ on the mode of bringing it about. To determine rightly on this important subject, it is essential to know: 1st. Of what description are the characters who are entrusted with the health of the mass of the people; 2dly, In what way the proper qualifications of that class of the faculty can be guaranteed to the public; and, 3dly, How uninformed persons can be prevented from acting in a capacity for which they are unfit. These questions are, I think, so easily answerable that any elaborate enquiry concerning them are unnecessary. General practitioners form the the great majority of the faculty, they have almost entirely superseded the apothecaries. The superior qualifications of general practitioners may be well guaranteed to the public, by obliging those gentlemen to become members of the Royal College of Surgeons before they are permitted to practise; and this will effect the exclusion of all those individuals, who, not being regularly educated, can never be equal to the performance of those duties connected with the station they usurp. General practitioners are the medical favourites of the community; the majority of persons having long wished for a class of the faculty to whom they could apply with confidence in any description of case in which medical and surgical aid was necessary. The English faculty, becoming, "like Virgil's army, so crowded that many of them had not room to use their weapons," found it necessary to practise the three branches of medicine together. The apothecary now began to practise physic and surgery. The surgeon, finding his province encroached upon by the apothecary, feeling the confidence of the public in his favour, and seeing the utility and convenience which must accrue to the community by practising the three branches of medicine, combined them in his own person.

Physic and surgery are by some still said to be distinct sciences, and many very unnatural and narrow-minded efforts have been made to render them so. Now a good surgeon (generally speaking) is the best anatomist; he well knows the doctrines and various treatment of inflammation, he is familiar with diseases, in the cure and palliation of which to chirurgical skill he combines the practice of medicine. "The internal and external parts of the body are governed by the same general laws during a state of health; and, if an internal part be attacked with inflammation, the appearances and effects will bear a great similarity to the same disease situated externally." And again, "the practical parts of physic and surgery are very frequently disunited, but their theory and principles are indivisible, for they truly constitute one and the same science."*

In an excellent Memoir on Medical Reform, by "A disinterested Physician," (Med. and Phys. Journ. vol. 30,) the preference evinced by the public in favour of the general practitioner is much dwelt upon; and it is therein said, that "The first attempt to profit by this experience, and to form a practitioner who should unite in his own person every department, with a fair presumption that he was duly qualified

* Preface to Pearson's Principles of Surgery, page 6.

in in all, was on the part of the London College of Surgeons, and in giving to the country the surgeon-apothecary;* familiarized to general practice by apprenticeship, grounded in a knowledge of the human body and of diseases in general, by attendance of lectures, dissections, and hospital practice; and *proved* as to his actual attainments, by examination; they have done more extensive and essential service to the community than all the other corporations united."†

To conclude, 1st. The community does not appear to me to wish or require any other classes of the faculty than physicians, surgeons, and general practitioners. 2dly, Surgeons, in my mind, are best fitted for general practice. 3dly, That, as the Royal College of Surgeons recognizes none others than those who have produced proper indentures, certificates of attendance to anatomy, physiology, surgery, hospital practice, &c. and who have submitted to an examination at the college on those subjects; we need not look to any other corporate body for approved surgeons or general practitioners. If what I have said is correct, it follows that the thoughts of government on medical reform should be exclusively directed to the College of Surgeons. The charter of the college should be extended; no man should be permitted to practise as surgeon or general practitioner without its diploma.‡ Here alone (except in regard to the charters of certain corporate bodies of physicians) will the interference of the legislature be salutary. In this way will

* Or rather the surgeon "familiarized to general practice," &c. for the college does not recognize the novel appellation of surgeon-apothecary.

† The College of Surgeons merits the warmest panegyric; its examinations are calculated to ensure to the public, gentlemen competent to the practice of the three branches of the profession; and this the public is convinced of by the experience it has for some years had of its members. Still, it must not be concealed, that, if those examinations were extended more immediately into the theory and practice of medicine and midwifery; and, above all, if the anatomical enquiries were instituted over the dead subject, well and fairly dissected, much good would arise. I very well recollect a pupil who, practically speaking, was extremely ignorant of anatomy, and yet who surprized his teacher with the facility he had acquired in answering the most difficult anatomical questions. Many anecdotes of this kind might be related. The above is, indeed, an important consideration, for, in the anatomist, we behold the able physician and surgeon.

‡ Since writing this paper, I rejoice to find the college introducing a bill into parliament to remedy some or all of those evils of which we complain.

a due a due supply of medical men well calculated to combine the three branches of medicine in their practice, be afforded to the public. All illiterate persons, "self-created surgeons," bone-setters, farriers, prescribing druggists and grocers, practising quacks, and ignorant women, will then no longer work miracles with their herbs, their balsams, and their tinctures; and the recurrence of "pain, mutilation, and death," will, in future, be oftener prevented. In short, an effectual barrier will be then placed against the admission of uninformed persons into the profession; a sufficient guarantee given to the public of the competency of all its members to the practice of medicine;—and then, and not till then, will that profession be enabled to acquire its proper dignity.

*Frome;* JOHN C. YEATMAN.
*July* 1, 1815.

